# Buffalo Medical College

**Published:** 1848-08

**Authors:** 


*Buffalo Medical College.*—The walls of the new edifice of the Medical Department of the University of Buffalo, are rapidly advancing. The structure is of stone—a red sand-stone, procured at Lockport, which has not before been used in this city for building purposes. The effect, so far as can be estimated from the present appearance of the walls, will be very fine. The building is one hundred feet deep, by about fifty feet front. It is located on Main Street a short distance above the Roman Catholic church. It is designed that the walls will be completed, and the building enclosed the present season.

